# Hepatic ChREBP orchestrates intrahepatic carbohydrate metabolism to limit hepatic glucose 6-phosphate and glycogen accumulation in a mouse model for acute Glycogen Storage Disease type Ib

**DOI:** 10.1016/j.molmet.2023.101838

**Published:** 2023-11-22

**Authors:** K.A. Krishnamurthy, M.G.S. Rutten, J.A. Hoogerland, T.H. van Dijk, T. Bos, M. Koehorst, M.P. de Vries, N.J. Kloosterhuis, H. Havinga, B.V. Schomakers, M. van Weeghel, J.C. Wolters, B.M. Bakker, M.H. Oosterveer

**Affiliations:** 1 Laboratory of Pediatrics, University of Groningen, University Medical Center Groningen, The Netherlands; 2 Department of Laboratory Medicine, University of Groningen, University Medical Center Groningen, The Netherlands; 3 Interfaculty Mass Spectrometry Center, University of Groningen, University Medical Center Groningen, The Netherlands; 4 Laboratory Genetic Metabolic Diseases, UMC Amsterdam, The Netherlands; 5 Core Facility Metabolomics, UMC Amsterdam, The Netherlands

**Keywords:** ChREBP, Glucokinase, Hepatic glycogen metabolism, Glycophagy, Glycogen Storage Disease type Ib, Stable isotope tracing

## Abstract

**Objective:**

Carbohydrate Response Element Binding Protein (ChREBP) is a glucose 6-phosphate (G6P)-sensitive transcription factor that acts as a metabolic switch to maintain intracellular glucose and phosphate homeostasis. Hepatic ChREBP is well-known for its regulatory role in glycolysis, the pentose phosphate pathway, and *de novo* lipogenesis. The physiological role of ChREBP in hepatic glycogen metabolism and blood glucose regulation has not been assessed in detail, and ChREBP's contribution to carbohydrate flux adaptations in hepatic Glycogen Storage Disease type 1 (GSD I) requires further investigation.

**Methods:**

The current study aimed to investigate the role of ChREBP as a regulator of glycogen metabolism in response to hepatic G6P accumulation, using a model for acute hepatic GSD type Ib. The immediate biochemical and regulatory responses to hepatic G6P accumulation were evaluated upon G6P transporter inhibition by the chlorogenic acid S4048 in mice that were either treated with a short hairpin RNA (shRNA) directed against ChREBP (shChREBP) or a scrambled shRNA (shSCR). Complementary stable isotope experiments were performed to quantify hepatic carbohydrate fluxes *in vivo*.

**Results:**

ShChREBP treatment normalized the S4048-mediated induction of hepatic ChREBP target genes to levels observed in vehicle- and shSCR-treated controls. In parallel, hepatic shChREBP treatment in S4048-infused mice resulted in a more pronounced accumulation of hepatic glycogen and further reduction of blood glucose levels compared to shSCR treatment. Hepatic ChREBP knockdown modestly increased glucokinase (GCK) flux in S4048-treated mice while it enhanced UDP-glucose turnover as well as glycogen synthase and phosphorylase fluxes. Hepatic GCK mRNA and protein levels were induced by shChREBP treatment in both vehicle- and S4048-treated mice, while glycogen synthase 2 (GYS2) and glycogen phosphorylase (PYGL) mRNA and protein levels were reduced. Finally, knockdown of hepatic ChREBP expression reduced starch domain binding protein 1 (STBD1) mRNA and protein levels while it inhibited acid alpha-glucosidase (GAA) activity, suggesting reduced capacity for lysosomal glycogen breakdown.

**Conclusions:**

Our data show that ChREBP activation controls hepatic glycogen and blood glucose levels in acute hepatic GSD Ib through concomitant regulation of glucose phosphorylation, glycogenesis, and glycogenolysis. ChREBP-mediated control of GCK enzyme levels aligns with corresponding adaptations in GCK flux. In contrast, ChREBP activation in response to acute hepatic GSD Ib exerts opposite effects on GYS2/PYGL enzyme levels and their corresponding fluxes, indicating that GYS2/PYGL expression levels are not limiting to their respective fluxes under these conditions.

## Highlights

• Hepatic ChREBP knockdown increases GCK levels and-flux.
• Hepatic ChREBP knockdown enhances hepatic glycogen synthesis and-phosphorylation in acute GSD Ib.
• Hepatic ChREBP knockdown reduces hepatic STBD1 levels and GAA activity.
• Hepatic ChREBP knockdown slightly reduces blood glucose levels in acute GSD Ib.

## Abbreviations

AGL: Glycogen debranching enzyme
ALDOB: Aldolase B
ChREBP: Carbohydrate Response Element Binding Protein
G6P: Glucose 6-Phosphate
G6PC: Glucose 6-Phosphatase
GAA: Acid maltase
GCK: Glucokinase
GCKR: Glucokinase Regulatory Protein
GSD I: Glycogen Storage Disease Type 1
GYS2: Glycogen Synthase 2
PKLR/L-PK: Liver Pyruvate Kinase
PPP: Pentose Phosphate Pathway
PYGL: Liver Glycogen Phosphorylase
shRNA: short hairpin RNA
SLC37A4: Solute Carrier 37A4
STBD1: Starch Binding Domain Protein 1
UDPglc: Uridine Di Phosphate glucose

## 1 Introduction

Carbohydrate Response Element Binding Protein (ChREBP) is a transcription factor that acts as a metabolic switch to maintain metabolic homeostasis in response to increased intracellular glucose availability [1]. Being one of the major metabolite sensors in hepatocytes, ChREBP is activated by different glucose-derived phosphate sugars through allosteric regulation and posttranslational modifications [[2], [3], [4]]. Consequently, both prevalent and rare metabolic diseases associated with increased intrahepatic glucose availability, such as type 2 diabetes and Glycogen Storage Disease type 1 (GSD I), are characterized by enhanced ChREBP activity in the liver [[5], [6], [7], [8], [9]]. Upon its activation, ChREBP induces the transcription of genes encoding key enzymes involved in glycolysis, the pentose phosphate pathway (PPP), and *de novo* lipogenesis (DNL). All these pathways consume glucose 6-phosphate (G6P), and enhanced ChREBP signaling has been proposed to contribute to the homeostatic control of intracellular glucose and phosphate [10].

Besides glycolysis, PPP and DNL, G6P is also used as a substrate for glycogen synthesis. Animal studies have shown that hepatic glycogen content is elevated upon ChREBP knockdown [9,11,12]. Moreover, ChIP-seq analysis of mouse liver tissue predicted ChREBP DNA binding at loci corresponding to glycogen synthase (*Gys2*) and glycogen phosphorylase (*Pygl*) [13], genes encoding hepatic enzymes that mediate glycogen synthesis and breakdown, hence consuming and producing hepatic G6P, respectively. Hepatic glycogen accumulation is emerging as a contributor to hepatopathy and liver tumor formation [[14], [15], [16], [17], [18]], while ChREBP has been proposed to play a pro-oncogenic role in the liver [16,19,20]. We previously reported that normalizing hepatic ChREBP activity, increased hepatic glycogen content and lowered blood glucose levels in hepatocyte-specific glucose-6-phosphatase (*G6pc*) knockout mice, a model for hepatic GSD type Ia [9,21,22]. These studies focused on hepatic lipid metabolism, liver disease progression, oncogenic signaling, and tumor susceptibility in a genetic model for hepatic GSD Ia, while the role of hepatic ChREBP in regulating glycogen metabolism was not addressed. Our previous research also showed that hepatic GSD Ia mice display G6PC-independent glucose production via alpha-glucosidase-dependent glycogen breakdown [23]. Thus, combined outcomes of previous studies suggest a role for ChREBP in regulating hepatic glycogen metabolism and/or G6PC-independent glucose production. Yet, the physiological function of ChREBP in hepatic glycogen metabolism and blood glucose regulation through regulation of carbohydrate fluxes has not yet been assessed. Such insight is essential to better understand the pathophysiology of metabolic diseases with liver involvement such as type 2 diabetes and GSD I, which are associated with constitutive activation of hepatic ChREBP, glucose, G6P and glycogen imbalances, and increased risk for hepatocellular transformation [24,25].

The current study aimed to investigate the role of ChREBP in mediating the initial adaptations in glycogen metabolism that occur in response to hepatic G6P accumulation, and to explore the mechanisms involved. For this purpose, we employed stable isotope methods to quantify hepatic glucose and glycogen fluxes in mice with either intact or attenuated hepatic ChREBP signaling, in which intrahepatic G6P levels were acutely increased by short-term infusion of the chlorogenic acid S4048, a pharmacological inhibitor of the G6P transporter SLC37A4 [26], which is deficient in GSD Ib patients. This approach allowed to investigate the role of hepatic ChREBP as a mediator of the immediate responses to hepatic G6P accumulation at physiological, biochemical, and regulatory levels.

## 2 Materials and methods

### 2.1 Animal experiments

**Figure 1:**
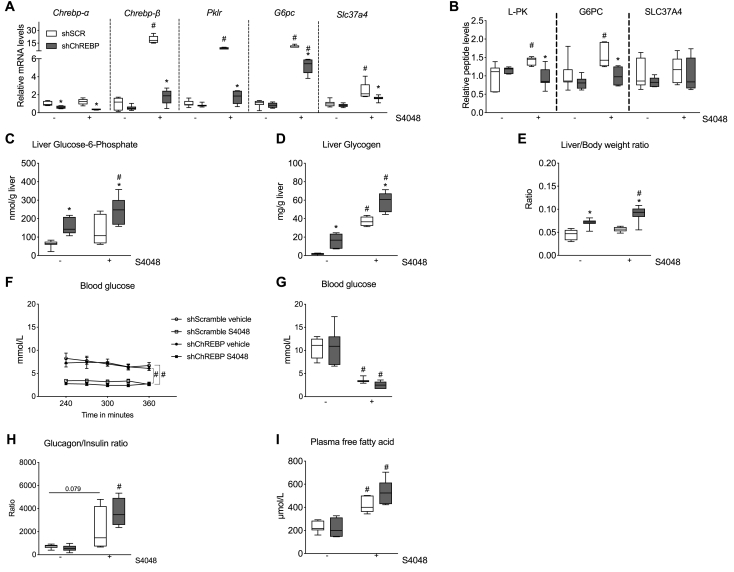
**Hepatic ChREBP knockdown aggravates hepatic glycogen accumulation and further reduces blood glucose levels in acute GSD Ib**. **A**. Hepatic *Chrebp-α, Chrebp-β, G6pc*, *Pklr*, and *Slc37a4* mRNA expression levels. **B**. Hepatic G6PC, L-PK and SLC37A4 protein levels determined by targeted proteomics. **C**. Hepatic glucose 6-phosphate contents. **D**. Hepatic glycogen contents. **E**. Relative liver weights. **F**. Steady-state (between 240 and 360 min) blood glucose levels during vehicle/S4048 infusion. **G**. Plasma glucose levels after 360 min of vehicle/S4048 infusion. **H**. Plasma glucagon-to-insulin ratios after 360 min of vehicle/S4048 infusion. **I**. Plasma free fatty acid levels after 360 min of vehicle/S4048 infusion. All panels present n = 5–7/group. For panels A-B, data are expressed relative to shSCR-treated mice infused with vehicle. The mRNA levels were normalized to β-actin. ∗*p* < 0.05 *vs.* shSCR receiving the same infusion (indicates a shChREBP effect). #*p* < 0.05 *vs.* vehicle receiving the same shRNA treatment (indicates an S4048 effect).

**Figure 2:**
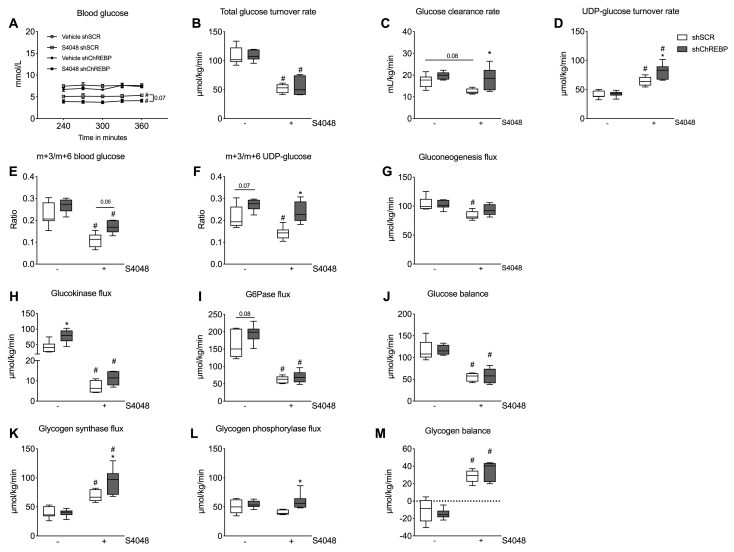
**Hepatic ChREBP knockdown enhances GCK and glycogen synthase fluxes and promotes UDP-glucose turnover in acute GSD Ib**. **A**. Steady-state (between 240 and 360 min) blood glucose levels during infusion of vehicle/S4048 and stable-isotopes. **B**. Total glucose turnover rates. **C**. Glucose clearance rates, calculated as total glucose turnover rates divided by the blood glucose concentrations. **D.** UDP-glucose turnover rates. **E.** m+3/m+6 ratios in blood glucose isolated from blood spots (n = 6–7/group). **F.** m+3/m+6 ratios in UDP-glucose isolated from urine samples. **G.** Gluconeogenesis flux. **H**. Glucokinase flux. **I**. G6Pase flux. **J**. Glucose balance (n = 6–7/group). **K**. Glycogen synthase flux. **L.** Glycogen phosphorylase flux. **M**. Glycogen balance. For all parameters, average values under isotopic steady-state conditions (between 240 and 360 min of infusion) are presented with n = 6–7/group. ∗*p* < 0.05 *vs.* shSCR receiving the same infusion (either vehicle or S4048). #*p* < 0.05 *vs.* vehicle receiving the same shRNA treatment (either shSCR or shChREBP).

**Figure 3:**
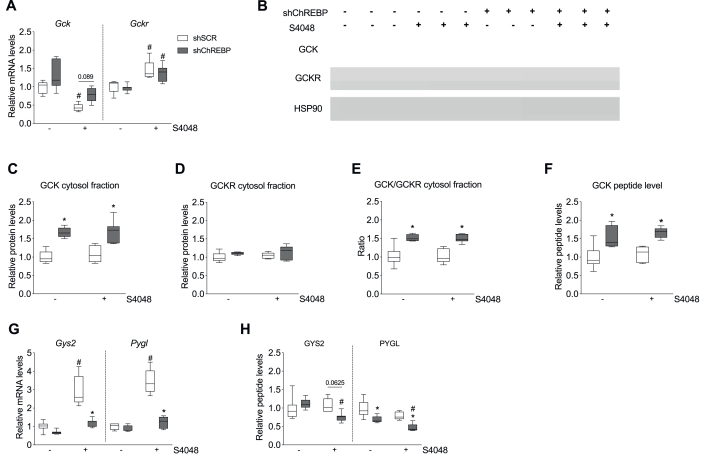
**Hepatic ChREBP knockdown induces GCK levels and reduces GYS2 and PYGL levels in acute GSD Ib**. **A**. Hepatic *Gck* and *Gckr* mRNA levels. **B**. Representative immunoblots of GCK, GCKR and HSP90 proteins in cytosolic liver fractions (n = 3/group). **C,D,E**. Quantification of cytosolic GCK and GCKR protein levels (n = 5–7/group). Values are normalized to HSP90, and expressed relative to the average level in shSCR-treated vehicle-infused mice (n = 5–7/group). **F**. Hepatic GCK protein levels determined by targeted proteomics (n = 5–7/group). **G.** Hepatic *Gys2* and *Pygl* mRNA levels (n = 6–7/group). **H.** Hepatic GYS2 and PYGL protein determined by targeted proteomics (n = 6–7/group). All data are expressed relative to shSCR-treated mice infused with vehicle. The mRNA levels were normalized to β-actin. ∗*p* < 0.05 *vs.* shSCR receiving the same infusion (either vehicle or S4048). #*p* < 0.05 *vs.* vehicle receiving the same shRNA treatment (either shSCR or shChREBP).

**Figure 4:**
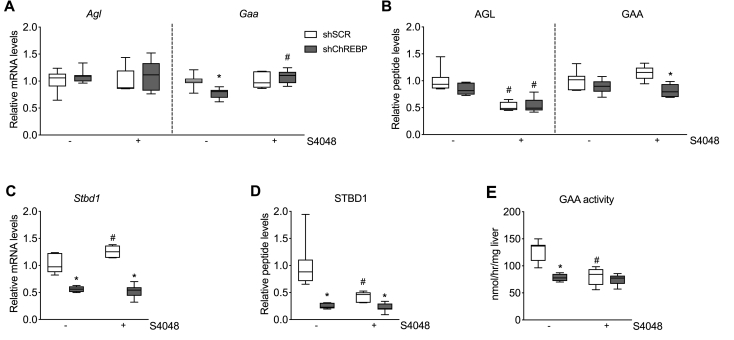
**Hepatic ChREBP knockdown reduces STBD1 levels and GAA activity in acute GSD Ib**. **A**. Hepatic *Agl* and *Gaa* mRNA levels. **B.** Hepatic AGL and GAA protein levels determined by targeted proteomics. **C.** Hepatic *Stbd1* mRNA levels. **D.** Hepatic STBD1 protein levels determined by targeted proteomics. **E.** Maximal enzymatic activity of GAA in liver homogenates. All panels present n = 6–7/group. For panels A-D, data are expressed relative to shSCR-treated mice infused with vehicle. The mRNA levels were normalized to β-actin. ∗*p* < 0.05 *vs.* shSCR receiving the same infusion (either vehicle or S4048). #*p* < 0.05 *vs.* vehicle receiving the same shRNA treatment (either shSCR or shChREBP).

Animal experiments were approved by the Institutional Animal Care and Use Committee of the University of Groningen (Groningen, The Netherlands), performed under CCD license number #AVD105002015245, in line with the Guide for the Care and Use of Laboratory Animals. Male adult C57BL/6J mice, aged 11 weeks, were housed individually in a light- and temperature-controlled facility (lights on from 7:00 AM to 7:00 PM). The animals were housed in individually ventilated cages and were provided with wood bedding, nesting material, and cardboard rolls (which were excluded upon placement of a jugular vein catheter). The animals had *ad libitum* access to drinking water and received standard laboratory chow diets, *i.e.*, RMH-B (Abdiets, Woerden, the Netherlands for the basal infusions experiment; Figure 1, Figure 3, Figure 4), or V1554-703; ssniff-Spezialdiäten GmbH, Soest, Germany for the stable isotope infusion experiment; Figure 2).

Animals were retro-orbitally injected with 5 × 10^12^ adeno-associated virus (AVV) particles per mouse containing a short hairpin RNA (shRNA) directed against ChREBP (shChREBP) or a scrambled control shRNA (shSCR) under isoflurane anesthesia. Construction and production of the viruses was performed as described [9]. Four weeks after injection, the mice were implanted with a permanent catheter in the jugular vein under isoflurane anesthesia and subsequently allowed to recover from surgery for 5–6 days. After recovery, one cohort of mice consisting of two groups injected with either shChREBP or shSCR were fasted overnight (10 PM-8 AM with drinking water available). Half of the mice from each group was subsequently infused for 6 h with S4048 (Sanofi-Aventis, Frankfurt, Germany; 5.5 mg/mL in sterile phosphate-buffered saline (PBS) with 6 % dimethyl sulfoxide (DMSO) pH 7.4 at an infusion rate of 0.135 mL/h) via the jugular vein catheter under continued fasting, while the other mice of each group received vehicle only (6 % DMSO in sterile PBS) at the same infusion rate. Blood glucose levels were measured every 30 min in tail blood using a handheld glucometer (Accu-Check, Roche, Mannheim, Germany). At the end of the 6-hour infusion period, animals were sacrificed under isoflurane anesthesia for blood and liver collection.

In order to quantify intrahepatic glucose fluxes, a second cohort of shChREBP- or shSCR-treated mice was overnight fasted and subsequently infused with S4048 or its vehicle for 6 h. Besides S4048 or vehicle, all four experimental groups received a sterile solution of stable isotopes, composed of D-[U–^13^C]-glucose (2.5 mg/mL), [2–^13^C]-glycerol (16 mg/mL), D-[1–^2^H]-galactose (6 mg/mL), and paracetamol (2 mg/mL) [27] at an infusion rate of 0.5 mL/h. Again, blood glucose levels were measured every 30 min in tail blood using a glucometer (Accu-Chek). In addition, blood spots from tail bleeding were collected every 60 min, and voluntary urine samples were collected at hourly intervals on filter paper. At the end of the 6-hour infusion period, animals were sacrificed under isoflurane anesthesia for blood and liver collection. Mice were excluded from the experiment during infusion, as their cannula was dislodged, blocked, or released, which occurred in three cases.

### 2.2 Isotopomer analyses and metabolic flux calculations

The enrichment of stably-labeled isotopes in both blood spots and urine samples was analyzed as described [23,26]. Briefly, urine samples were extracted from filter paper using methanol, followed by the conversion of paracetamol-glucuronic acid to paracetamol glucoside by overnight treatment with sodium borohydride. Subsequently, paracetamol glucoside was isolated through HPLC fractionation and samples were incubated with β-glucosidase to liberate paracetamol and free glucose. Blood spots collected on filter paper were incubated with 50 μL of Milli-Q water for 15 min at RT extract glucose. Then, 500 μL of ethanol was added and samples were incubated overnight. Finally, the solution was centrifuged for 10 min at 14000 rpm, after which 200 μL of the supernatant was used for further analysis. Urinary and blood glucose samples were subsequently subjected to glucose pentaacetate and glucose aldonitril pentaacetate derivatization after which samples were analyzed by gas chromatography-mass spectrometry (Agilent 9575C inert MSD; Agilent tech, Amstelveen, The Netherlands) as described [26]. The data were corrected for the natural abundance of ^13^C as described previously [28]. Based on the isotopomer distributions, carbohydrate fluxes were calculated as described [26] and averaged over the isotopic steady state period, *i.e.*, 240–360 min after the start of the isotope infusion. A scheme of the experimental approach and an overview of the mathematical equations used for hepatic carbohydrate flux calculations are presented in Supplementary Figure 1.

### 2.3 qPCR analysis

RNA was extracted from liver tissue using TriZol reagent (Sigma–Aldrich, St. Louis, MO). Complementary DNA (cDNA) was obtained using reverse transcriptase (M-MLV 28025013, Invitrogen, Waltham, MA) according to the manufacturer's instructions, and cDNA was subsequently amplified either using SYBR Green or Taqman probes using the primers and probes listed in Supplementary Table 1. mRNA levels were quantified based on a dilution curve generated from a pool of all samples, expressed relative to β-actin mRNA levels, and normalized to the average expression level of shSCR-treated vehicle-infused mice.

### 2.4 Immunoblot analysis

Cytosolic and nuclear fractions of liver tissue were prepared using the NE-PER Nuclear and Cytoplasmic Extraction Reagents (ThermoFischer #78833) according to the manufacturer's protocol, with the adaptation that the tissue was homogenized using a Precellys Evolution bead-beating homogenizer (Bertin technologies) at 6000Hz, 2 × 15 s with a 30 s break in between. Protein concentrations of the cytosolic and nuclear fractions were quantified using the BCA protein quantification method (BCA Protein Assay Kit, ThermoScientific, #23225). For SDS-PAGE, sample volumes corresponding to 30 μg of isolated protein were loaded and separated on 10 % polyacrylamide gels. The proteins on the gels were subsequently transferred to PVDF membranes (GE Healthcare Life Science, #A29574727, AmershamTM HybondTM) using wet/tank blotting for 2 h at 45V. The membranes were then blocked with 5 % BSA solution in TBST (10 mmol/L Tris–HCl pH 8.0, 150 mmol/L NaCl, and 0.1 % Tween 20) for 1 h at room temperature, and subsequently incubated with primary antibodies (Supplementary Table 2) prepared in 5 % BSA in TBST overnight at 4 °C.

On the next day, the membranes were washed with TBST 3 times for 10 min at room temperature and incubated with a matching HRP-tagged secondary antibody (Supplementary Table 2) for 1 h at room temperature. After this, the membranes were again washed were washed with TBST 3 times for 10 min at room temperature. Finally, they were incubated with ECL substrate (ThermoFischer scientific #32106) for 5 min and imaged using a GE imager, ImageQuant LAS 4000 mini machine (Cytiva, GE Healthcare). Multiple blots from gels ran in parallel were quantified using ImageQuant TL software (version 8.2.0, General Electric Company), and band intensities were normalized to HSP90 signals for cytosolic fractions, and to Lamin A/C signals for nuclear fractions, and subsequently expressed relative to values of shSCR-treated vehicle-infused mice.

### 2.5 Targeted proteomics

Targeted proteomics was performed on delipidated liver homogenates using isotopically labeled peptide standards (Supplementary Table 3) as described [9]. The concentrations were related to the total peptide content, which was determined by a colorimetric peptide assay after tryptic digestion and SPE cleanup (Thermo Scientific#23275). Endogenous peptide amounts were calculated from the known concentration of the labeled standards and expressed in fmol peptide per μg total protein. The calculated peptide amounts were subsequently expressed relative to amounts of shSCR-treated vehicle-infused mice.

### 2.6 Metabolite and hormone analyses

Analysis of plasma lactate (#2864 Instruchemie Delfzijl, The Netherlands), ketone body (#415–73301 FUJIFILM Wako Chemicals Europe GmbH, Neuss, Germany) and free fatty acid levels (#157819910935 Holzheim, Germany) was performed as described [9]. Plasma insulin and glucagon levels were analyzed using an ELISA kit (#90095 and #81520 Crystal Chem Europe, The Netherlands). G6P and glycogen contents in liver samples were quantified as previously described [29].

Metabolomics was performed for the liver samples using Liquid Chromatography coupled to Mass Spectrometry as described previously [30], and metabolite levels were expressed relative to the shSCR-treated vehicle-infused mice.

### 2.7 Acid alpha-glucosidase activity analysis

Maximal acid alpha-glucosidase (GAA) activity from liver homogenates was quantified as described [31], using glycogen as a substrate. Data were normalized to protein content (BCA Protein Assay Kit, ThermoScientific, #23225) and expressed as nmol/hour/mg protein.

### 2.8 Statistical analysis

Data in figures are presented as min–max box plots and the data in tables is presented as mean values with standard error (SE). Differences between groups were analyzed using a two-way ANOVA, followed by Tukey post-hoc pairwise comparisons using GraphPad Prism version 9.5 (GraphPad Software Inc., La Jolla, CA) using a significance level of *p* < 0.05. Results of the post-hoc comparisons are indicated in Tables and Figures, with ∗ marking statistically significant effects of shChREBP treatment within groups receiving the same infusion (either vehicle or S4048), and # marking statistically significant effects of S4048 infusion within groups treated with the same shRNA (either shSCR or shChREBP).

## 3 Results

### 3.1 Hepatic ChREBP knockdown aggravates hepatic glycogen accumulation and further reduces blood glucose levels in acute GSD Ib

To investigate the role of ChREBP in hepatic glycogen metabolism, mice were treated with a shRNA directed against ChREBP (shChREBP) or a scrambled control shRNA (shSCR), and subsequently received a short-term (6 h) intravenous infusion with S4048 to induce acute hepatic GSD Ib, or with its vehicle. Validation of hepatic *Chrebp* knockdown by qPCR revealed that shChREBP treatment significantly reduced *Chrebp-α* mRNA expression in vehicle- (40 %) and S4048-infused (65 %) mice (Figure 1A). Hepatic mRNA levels of *Chrebp-β*, liver pyruvate kinase *(Pklr)*, *G6pc*, and *Slc37a4* and protein levels of G6PC and liver pyruvate kinase (L-PK) were comparable between shSCR- and shChREBP-treated mice infused with vehicle, while the S4048-mediated induction of these mRNAs and proteins was attenuated by shChREBP treatment as compared to shSCR treatment (Figure 1A**-**1B). SLC37A4 protein levels remained unaffected by any treatment (Figure 1B). Because these findings indicate that shChREBP treatment normalized ChREBP target gene expression levels in S4048-infused mice to vehicle-infused levels, this approach was used further to assess the role of ChREBP in hepatic glucose and glycogen metabolism.

shChREBP-treated mice infused with vehicle showed significantly higher hepatic G6P and glycogen levels compared to their shSCR-treated controls (Figure 1C and D). Additionally, S4048 infusion increased hepatic G6P and glycogen contents in shSCR-treated mice, and these increments were exacerbated by shChREBP treatment (Figure 1C and D). Furthermore, mass spectrometry analysis confirmed that hepatic levels of G6P, and of other hexose phosphates, were elevated by both S4048 infusion and shChREBP treatment (Table 1). Intrahepatic free glucose contents, on the other hand, were significantly reduced in shChREBP-treated mice infused with S4048 as compared to vehicle-infused shChREBP-treated controls (Table 1). Among intermediates of glycolysis, hepatic fructose 1,6-bisphosphate (F1,6BP) levels were slightly but non-significantly increased in S4048 -infused mice, while they were unaffected by shChREBP treatment compared to shSCR-treated controls (Table 1). The level of 3-phosphoglyceric acid (3 PG) was significantly reduced by S4048 infusion in shSCR-treated mice, as well as by shChREBP treatment in vehicle-infused mice. The combination of S4048 and shChREBP normalized 3 PG towards levels in vehicle-infused shSCR-treated mice (Table 1). 2-phosphoglyceric acid showed the same pattern, but differences were not significant. Hepatic phosphoenolpyruvate (PEP) and lactate levels were increased by S4048 infusion in shSCR-treated mice, and normalized towards shSCR-vehicle levels by shChREBP treatment in S4048-infused mice. Pyruvate levels remained unaffected by S4048 infusion in shSCR-treated mice, but were slightly reduced in shChREBP-treated S4048-infused mice as compared to shChREBP-treated vehicle-infused controls (Table 1). Among pentose phosphate pathway intermediates, hepatic ribose levels were significantly elevated by S4048 infusion in shSCR-treated mice compared to vehicle-infused controls, while they were reduced by shChREBP treatment in vehicle-infused mice, and normalized towards levels in vehicle-infused shSCR-treated mice by the combination of S4048 and shChREBP (Table 1). Hepatic sedoheptulose content was significantly elevated upon S4048 infusion in both shSCR- and shChREBP-treated mice. Moreover, sedoheptulose 7-phosphate levels were significantly increased by S4048 infusion in shSCR-treated mice and moderately increased in shChREBP-treated mice. S4048 infusion also significantly increased hepatic gluconate 6-phosphate, 2-dehydrogluconate 6-phosphate, and ribose 5-phosphate, while shChREBP treatment alleviated these increments in S4048-infused mice (Table 1), indicating a ChREBP-mediated accumulation of these pentose phosphate intermediates in S4048 infused mice. The S4048- and shChREBP-mediated changes in G6P, hexose phosphates, F16BP, pyruvate, lactate, ribose 5-phosphate, and sedoheptulose 7-phosphate are in line with our previous findings in hepatocyte-specific *G6pc* knockout and shChREBP-treated mice [9]. Altogether, S4048 infusion increased the hepatic content of specific glycolytic and pentose phosphate pathway intermediates while shChREBP treatment alleviated the accumulation of gluconate 6-phosphate, 2-dehydrogluconate 6-phosphate, ribose 5-phosphate, and glycogen in the liver of S4048-infused mice.

**Table 1:** Hepatic metabolite contents.

Hepatic metabolite level (Mean ± SEM)	shSCR vehicle	shChREBP vehicle	shSCR S4048	shChREBP S4048
Glucose	1.0 ± 0.1	1.3 ± 0.1	1.0 ± 0.2	0.7 ± 0.0∗
Glucose 6-Phosphate	1.0 ± 0.2	4.8 ± 0.4∗	3.3 ± 0.5^#^	3.6 ± 0.3
Hexose Phosphates^a^	1.0 ± 0.4	4.9 ± 0.6∗	5.3 ± 1.0^#^	4.9 ± 2.4
Fructose 1,6-Bisphosphate	1.0 ± 0.2	1.2 ± 0.1	1.9 ± 0.3	2.3 ± 0.4
2-Phosphoglyceric Acid	1.0 ± 0.1	0.8 ± 0.0	0.8 ± 0.1	1.1 ± 0.1
3-Phosphoglyceric Acid	1.0 ± 0.1	0.7 ± 0.0∗	0.6 ± 0.0^#^	0.9 ± 0.1
Phosphoenol Pyruvate	1.0 ± 0.2	1.4 ± 0.1	1.8 ± 0.3	1.1 ± 0.1
Pyruvate	1.0 ± 0.1	1.3 ± 0.1	1.0 ± 0.1	0.8 ± 0.1^#^
Lactate	1.0 ± 0.1	1.2 ± 0.1	1.6 ± 0.2^#^	1.4 ± 0.1
Gluconate	1.0 ± 0.2	1.5 ± 0.2	1.3 ± 0.2∗^#^	0.9 ± 0.2
Gluconate 6-Phosphate	1.0 ± 0.2	2.9 ± 0.4	6.6 ± 1.3^#^	3.6 ± 0.7
2-Dehydrogluconate	1.0 ± 0.1	0.9 ± 0.1	1.1 ± 0.2	0.4 ± 0.1∗
2-Dehydrogluconate 6-Phosphate	1.0 ± 0.1	2.9 ± 0.4∗	2.4 ± 0.3^#^	1.3 ± 0.2^#^
Ribose	1.0 ± 0.1	1.5 ± 0.2	2.0 ± 0.3^#^	1.5 ± 0.3
Ribose 5-Phosphate	1.0 ± 0.1	1.7 ± 0.1	2.1 ± 0.4^#^	0.9 ± 0.1∗
Sedoheptulose	1.0 ± 0.1	1.7 ± 0.1	5.2 ± 1.0^#^	4.9 ± 0.9^#^
Sedoheptulose 7-Phosphate	1.0 ± 0.4	1.2 ± 0.2	2.9 ± 0.4^#^	3.7 ± 0.7

Data is normalized to levels in shSCR-treated infused with vehicle (n = 5–7/group).

∗*p* < 0.05 *vs.* shSCR receiving the same infusion (either vehicle or S4048).

#*p* < 0.05 *vs.* vehicle receiving the same shRNA treatment (either shSCR or shChREBP).

^a^ Hexose Phosphates reflects the sum of Galactose 1-Phosphate, Glucose 1-Phosphate, Fructose 1-Phosphate, Fructose 6-Phosphate, and Mannose 6-Phosphate, which cannot be distinguished by the Mass Spectrometry approach used.

shChREBP-treated mice infused with either vehicle or S4048 showed significantly higher liver-to-body weight ratios than their respective shSCR-treated controls, while relative liver weights were similar between vehicle- and S4048-infused shSCR-treated mice (Figure 1E and Table 2). Furthermore, during steady-state glycemic conditions (*i.e.*, between 240 and 360 min of infusion), blood glucose levels were significantly reduced in S4048-infused mice compared to their respective vehicle-infused controls (Figure 1F). As we repetitively observed that blood glucose levels in a subset of shChREBP-treated mice infused with S4048 reached the detection limit of the glucometer (*i.e.*, 1.1 mmol/L), glucose concentrations in plasma samples collected at the end of the infusion (*i.e.*, after 6 h) were also analyzed enzymatically. Plasma glucose analysis showed a tendency towards lower glucose levels in shChREBP-treated mice infused with S4048 compared to shSCR-treated S4048-infused mice (*p* = 0.85, Figure 1G). Consistent with this finding, shChREBP-treated/S4048-infused mice showed significantly increased plasma glucagon-to-insulin ratios and free fatty acid and ketone body levels compared to shSCR-treated/S4048-infused controls at the end of the infusion period (Figure 1H, I and Table 1). As expected, plasma glucagon-to-insulin ratios and free fatty acid and ketone body levels were higher in S4048-infused groups than in their vehicle-infused controls (Figure 1H and I, Table 1), while plasma lactate levels were comparable between groups (Table 2).

**Table 2:** Liver and plasma parameters.

	shSCR vehicle	shChREBP vehicle	shSCR S4048	shChREBP S4048
Body weight (grams)	21.8 ± 0.5	21.4 ± 0.4	22.0 ± 0.7	21.7 ± 0.6
Liver weight (grams)	0.97 ± 0.1	1.51 ± 0.1∗	1.20 ± 0.05	1.94 ± 0.1∗^#^
Plasma lactate (mmol/L)	1.5 ± 0.2	1.5 ± 0.1	1.4 ± 0.2	1.1 ± 0.2
Plasma ketone bodies (mmol/L)	0.7 ± 0.04	0.8 ± 0.2	1.4 ± 0.2^#^	1.9 ± 0.2^#^
Plasma insulin (ng/mL)	0.20 ± 0.01	0.204 ± 0.03	0.163 ± 0.02	0.074 ± 0.01^#^
Plasma glucagon (pg/mL)	144.5 ± 15.3	133.2 ± 10.0	234.7 ± 34.4^#^	281.8 ± 23.9^#^

Data is presented as mean ± SEM (n = 5–7/group).

∗*p* < 0.05 *vs.* shSCR receiving the same infusion (either vehicle or S4048).

#*p* < 0.05 *vs.* vehicle receiving the same shRNA treatment (either shSCR or shChREBP).

These findings indicate that shChREBP treatment in acute hepatic GSD Ib increases hepatic G6P and glycogen accumulation in parallel to hypoglycemia-associated changes in plasma glucagon-to-insulin ratios, and corresponding changes in free fatty acid and ketone body levels.

### 3.2 Hepatic ChREBP knockdown increases GCK flux and enhances hepatic glycogen metabolism in acute GSD Ib

Next, we investigated the mechanisms contributing to increased hepatic glycogen content and lower blood glucose levels in shChREBP-treated mice infused with S4048. For this purpose, a separate cohort of mice received stable-isotope tracer infusions along with vehicle or S4048, allowing to quantify intrahepatic glucose fluxes *in vivo* [27]. Specifically, the metabolic tracers D-[U–^13^C]-glucose, [2–^13^C]-glycerol, D-[1–^2^H]-galactose were simultaneously infused along with paracetamol (Supplementary Figure 1). Under isotopic steady-state conditions (240–360 min after the start of the infusion), dilution of the infused isotopes and isotope exchange rates in blood glucose and urinary (UDP-) glucose were calculated (Table 3). shChREBP-treated mice infused with S4048 consistently showed lower blood glucose levels than their shSCR-treated controls during the course of isotope infusion (Figure 2A). In line with these data, total glucose turnover rates were lowered by S4048 infusion (Figure 2B), while they remained unaffected by shChREBP treatment (Figure 2B). Glucose clearance rates were reduced in shSCR-treated mice infused with S4048 and normalized by shChREBP treatment (Figure 2C). S4048 infusion significantly increased UDP-glucose turnover rates, which was exacerbated by shChREBP treatment (Figure 2D). S4048 infusion reduced m+3/m+6 ratios in blood- and UDP-glucose in shSCR-treated mice as compared to vehicle-infused controls, while these ratios were increased by shChREBP treatment, particularly in S4048-infused mice (Figure 2E and F). As m+3 isotopomers in blood and UDP glucose originate from the infused uniformly labeled (m+6) glucose during glycolysis, the observed changes in m+3/m+6 ratios likely reflect reduced and increased contributions of glycolytic intermediates for glucose and glycogen production [32] in response to S4048 and shChREBP, respectively. This is consistent with S4048 enhancing glycolysis and shChREBP inhibiting lower glycolysis. The gluconeogenic flux was significantly decreased by S4048 infusion in shSCR-treated mice (Figure 2G). However, the effect was partly restored in shChREBP-treated mice infused with S4048 (Figure 2G). S4048 treatment reduced the glucokinase (GCK) flux, while GCK flux was significantly higher in shChREBP- compared to shSCR-treated mice receiving vehicle infusion (Figure 2H). As expected, S4048 reduced the glucose 6-phosphatase (G6Pase) flux, while G6Pase flux tended to be increased in vehicle-infused shChREBP- *vs.* shSCR-treated mice (Figure 2I). The hepatic glucose balance was similarly reduced by S4048 infusion in shSCR- and shChREBP-treated mice, while it was not affected by shChREBP treatment (Figure 2J). S4048 increased glycogen synthase (GSY2) flux in shSCR-treated mice compared to vehicle-treated controls, an effect that was exacerbated by shChREBP treatment (Figure 2K). On the other hand, glycogen phosphorylase (PYGL) flux remained unaffected by S4048 infusion but was increased in shChREBP *vs*. shSCR-treated mice infused with S4048 (Figure 2L). The hepatic glycogen balance was increased by S4048 infusion in shSCR- and shChREBP-treated mice (Figure 2M). Thus, *in vivo* flux analysis showed that shChREBP treatment directed intrahepatic glucose metabolism towards enhanced glycogen metabolism and GCK flux in hepatic GSD Ib.

**Table 3:** Primary isotope parameters during steady state (i.e. 240 and 360 min) infusion.

	shSCR vehicle	shChREBP vehicle	shSCR S4048	shChREBP S4048
Isotope dilution				
d(glc)	0.033 ± 0.0022	0.031 ± 0.0011	0.064 ± 0.0044^#^	0.059 ± 0.0035^#^
d(UDPglc)	0.262 ± 0.0095	0.242 ± 0.0129	0.161 ± 0.007^#^	0.123 ± 0.009^#^
Isotope exchange				
c(glc)	0.215 ± 0.017	0.3211 ± 0.02∗	0.0562 ± 0.0088^#^	0.0743 ± 0.0078^#^
c(UDPglc)	0.188 ± 0.011	0.186 ± 0.003	0.201 ± 0.007	0.233 ± 0.014^#^
MIDA				
f(glc)	0.786 ± 0.010	0.751 ± 0.004	0.756 ± 0.011	0.711 ± 0.013∗
f(UDPglc)	0.6651 ± 0.0075	0.6498 ± 0.0094	0.7519 ± 0.0109^#^	0.7456 ± 0.0044^#^

-MIDA: mass isotopomer distribution analysis. - d(glc): dilution of infused labelled glucose (D-[U–^13^C]-glucose) in pool of blood glucose. - d(UDPglc): dilution of infused labelled galactose (D-[1–^2^H]-galactose) in pool of UDP-glucose. - c(glc): fractional contribution of blood glucose in UDP-glucose (ratio of measured fractional isotopologue enrichment of U–^13^C-labelled glucose as measured in the glucose part of UDP-glucose over the fractional enrichment measured in blood glucose). - c(UDPglc): fractional contribution of UDP-glucose in blood glucose (ratio of fractional isotopologue enrichment of 1-^2^H-labelled blood glucose over the fractional enrichment measured in the glucose part of UDP-glucose). - f(glc): fractional contribution of gluconeogenesis (fructose-1,6-bisphosphate (F16P)) in blood glucose (ratio of measured fractional isotopologue enrichment of doubly-labelled blood glucose over the theoretical isotopologue enrichment of newly synthesized doubly-labelled F16P∗ as calculated by MIDA). -f(UDPglc): fractional contribution of gluconeogenesis (F16P) in UDP-glucose (ratio of measured fractional isotopologue enrichment of doubly-labelled glucose as measured in the glucose part of UDP-glucose over the theoretical isotopologue enrichment of newly synthesized doubly -labelled F16P∗ as calculated by MIDA). ∗*As doubly-labelled F16P arises from two monomers of [2-*^*13*^*C]-glycerol, the [2-*^*13*^*C]-glycerol infused is the responsible for double labelling of F16P. As such, [2-*^*13*^*C]-glycerol infusion directly relates to the calculation of parameters f(glc) and f(UDPglc) in the flux balance model applied.*

Data is presented as a mean with their respective SEM (n = 6–7/group).

∗*p* < 0.05 *vs.* shSCR receiving the same infusion (either vehicle or S4048).

#*p* < 0.05 *vs.* vehicle receiving the same shRNA treatment (either shSCR or shChREBP).

### 3.3 Hepatic ChREBP knockdown increases hepatic GCK and reduces GYS2 and PYGL expression in acute GSD Ib

To investigate the mechanism underlying altered GCK fluxes and UDP-glucose recycling in shChREBP-treated mice, we quantified hepatic mRNA and protein levels of relevant enzymes and other proteins. Hepatic mRNA levels of *Gck*, the hexokinase isoenzyme predominantly contributing to the glucokinase flux, were induced in shChREBP- *vs*. shSCR-treated mice, while S4048 infusion reduced *Gck* expression compared to vehicle (Figure 3A). Thus, *Gck* mRNA levels qualitatively showed the same patterns as GCK fluxes. On the other hand, mRNA levels of GCKR (Glucokinase Regulatory Protein), a negative regulator of GCK activity*,* were induced by S4048 infusion but unexpectedly [10] remained unaffected by shChREBP treatment (Figure 3A). Immunoblot analysis showed that cytosolic GCK protein levels were increased in the livers of shChREBP-treated mice infused with vehicle or S4048 (Figure 3B–C). Cytosolic GCKR protein levels were comparable in all groups (Figure 3D), whereas cytosolic GCK-to-GCKR ratios were increased in the livers of shChREBP-treated mice infused with vehicle or S4048 (Figure 3E). This finding was confirmed by complementary targeted proteomic analysis, which showed increased GCK protein content in whole liver lysates in shChREBP- *vs*. shSCR-treated mice (Figure 3F). Nuclear GCK-to-GCKR ratios were unaffected both by shChREBP treatment and S4048 infusion compared to the respective controls (Supplementary Figure 2).

The mRNA levels of *Gys2* and *Pygl,* which relate to glycogen synthase and -phosphorylase fluxes respectively*,* were increased by S4048 infusion and normalized by shChREBP treatment (Figure 3G). Targeted proteomic analysis revealed that GYS2 protein levels were comparable between the groups, while PYGL protein levels were reduced by shChREBP treatment and remained unaffected by S4048 infusion (Figure 3H).

Altogether, enhanced GCK flux in shChREBP-treated mice associates with increased GCK mRNA and protein levels and unaltered GCKR levels. In contrast and intriguingly, enhanced glycogen synthase flux and glycogen cycling in shChREBP-treated hepatic GSD Ib mice is paralleled by lower, rather than higher, GYS2 and PYGL levels.

### 3.4 Hepatic STBD1 expression and maximal GAA activity are reduced upon hepatic ChREBP knockdown and in response to acute GSD Ib

Besides cytosolic glycogen breakdown to G6P, hepatic glycogen can be converted into free glucose via two pathways, i.e., cytosolic glycogen debranching enzyme (AGL) or by lysosomal acid alpha-glucosidase (GAA), enabling G6PC-independent glucose production [23]. In order to assess whether these pathways are regulated by hepatic ChREBP and are associated with the changes in hepatic glycogen content and glycemia in shChREBP-treated mice, we quantified the levels of these enzymes. mRNA levels of AGL were comparable between groups while GAA mRNA levels were unaffected with S4048 infusion compared to shSCR-treated mice infused with vehicle (Figure 4A). shChREBP treatment in vehicle-infused mice showed significantly reduced *Gaa* expression, while this effect was normalized by S4048 infusion (Figure 4A). Yet, targeted proteomic analysis showed that AGL protein levels were significantly reduced in S4048-infused mice compared to vehicle-infused controls and unaffected by shChREBP treatment, while GAA protein levels were decreased by shChREBP treatment in S4048-infused mice (Figure 4B). mRNA levels of STBD1, which encodes an anchoring protein that targets glycogen to the lysosomes in hepatocytes [33], were induced by S4048 infusion in shSCR-treated mice and were reduced by shChREBP treatment in both vehicle- and S4048-infused mice (Figure 4C). In parallel, STBD1 protein levels (Figure 4D) and maximal GAA enzymatic activity (*V*_*max*_ in liver extract, Figure 4E) were found to be reduced by both shChREBP and S4048 treatment as compared to their respective controls.

Altogether, these data indicate that S4048 infusion and shChREBP treatment independently reduce hepatic STBD1 levels and maximal GAA enzymatic activity. Interestingly, the shChREBP- and S4048-mediated reductions in maximal GAA activity closely resembled the concomitant changes in STBD1 protein level, rather than shChREBP- and S4048-mediated effects on GAA mRNA or protein levels.

## 4 Discussion

This study aimed to investigate the role of hepatic ChREBP as a mediator of the immediate responses to hepatic G6P accumulation at physiological, biochemical, and regulatory levels in a mouse model for acute hepatic GSD Ib. Our data reveal unexpected and original functions of ChREBP in the liver. Hepatic ChREBP knockdown reduced glycogen synthase and -phosphorylase expression, yet, the respective fluxes through these pathways were found to be increased in ChREBP-normalized GSD Ib mice. At the same time, the expression of STBD1 and maximal activity of the lysosomal acid maltase, GAA, key players in lysosomal glycogen breakdown, were reduced upon ChREBP knockdown in GSD Ib mice. Interestingly, hepatic ChREBP knockdown increased GCK mRNA and protein levels and enhanced glucokinase flux. These changes were associated with a further accumulation of hepatic G6P and glycogen and a small decrease in blood glucose levels in ChREBP-normalized GSD Ib mice compared to GSD Ib mice. Our results show that hepatic ChREBP activation in hepatic GSD Ib serves to limit G6P and glycogen accumulation, while it simultaneously contributes to blood glucose control. These findings align with the previously proposed concept that hepatic ChREBP carries out a protective function in metabolically compromised states [1]. Yet, although the observed ChREBP-dependent adjustments in hepatic enzyme levels in acute hepatic GSD Ib likely serve to direct G6P toward glycogenesis and glucose production, these adjustments not necessarily translate into corresponding adaptions in the respective fluxes. Notably, while increased GCK levels in livers of ChREBP-normalized hepatic GSD Ib mice aligned with a higher GCK flux, lower GYS2 and PYGL levels associated with increased fluxes through these enzymes and enhanced glycogenesis. These findings indicate that GYS2 and PYGL levels were not limiting to the respective fluxes, and that metabolite and/or hormone-dependent regulation dominated in ChREBP-normalized hepatic GSD Ib mice.

S4048 treatment enhanced glycogen synthase flux and did not affect glycogen phosphorylase flux, while the mRNA expression of GYS2 and PYGL were induced and their protein levels remained unaltered. These flux data are consistent with a previous study from our laboratory in S4048-infused rats [26]. On the other hand, they deviate from our more recent study in hepatocyte-specific *G6pc* knockout mice, which showed an increase in both glycogen synthase and -phosphorylase fluxes compared to wildtype controls [23]. This discrepancy in glycogen phosphorylase flux in response to acute GSD Ib versus prolonged genetic GSD Ia likely relates to the duration of G6P accumulation (*i.e.*, hours versus days-weeks). Alternatively, differential cellular compartmentation of accumulated G6P, with predominant cytoplasmic accumulation in S4048-treated mice, and concomitant endoplasmic reticulum and cytosolic accumulation in hepatocyte-specific *G6pc* knockout mice, may contribute to this difference. The observation that S4048-mediated changes in mRNA expression levels did not always translate into changes in the respective protein levels during the course of the infusion experiment may be related to specific protein turnover rates. In agreement with molecular studies suggesting ChREBP-dependent regulation of *Gys2* and *Pygl* [13], we observed that the S4048-mediated induction of these genes and their protein levels was attenuated in shChREBP-treated mice. While GYS2 and PYGL levels were reduced by shChREBP treatment in S4048-infused mice, the fluxes through these enzymes were increased. This suggests that metabolite or hormone-driven regulation of glycogen synthase and -phosphorylase likely dominates, and that reduced GYS2 and PYGL levels in shChREBP-treated mice were not limiting the respective fluxes through these enzymes. In shChREBP-treated mice infused with S4048, m+3/m+6 ratios in blood glucose and urinary UDP-glucose were higher, suggesting higher contributions of glycolytic intermediates for glycogen and glucose production [32], and consistently, GCK levels and -flux were increased. These adaptations mark a redirection of intrahepatic metabolism towards G6P synthesis in shChREBP-treated S4048-infused mice, which may allosterically activate glycogen synthase [34], thereby further enhancing hepatic glycogen accumulation in shChREBP-treated S4048-infused mice.

In parallel to enhanced glycogen synthase flux, hepatic STBD1 and GAA protein levels and maximal hepatic GAA enzymatic activity were significantly reduced upon ChREBP normalization in acute hepatic GSD Ib. It has been well-established that STBD1 mediates translocation of glycogen to the lysosomes [33]. Interestingly, shChREBP treatment significantly reduced *Stbd1* mRNA expression in both vehicle- and S4048-infused mice, suggesting that *Stbd1* is transcriptionally regulated by ChREBP. In support of this, consultation of the Eukaryotic Promoter Database (https://epd.epfl.ch/index.php) and computational analysis revealed putative ChREBP binding sites (CACGTG; https://jaspar.genereg.net/) in the mouse and human *Stbd1* promotors. Moreover, hepatic ChREBP overexpression induces Stbd1 mRNA levels in mice (Gene expression omnibus series - 61576) [35]. On the other hand, mouse liver ChIP-seq data only showed a minor association of ChREBP on the Stbd1 promoter region [13]. These findings indicate that the transcriptional regulation of Stbd1 by ChREBP warrants in-depth molecular investigation. Previous work showed unaltered [36] or lower [37] hepatic glycogen content in *Stbd1* knockout mice. Additionally, *Stbd1*/*Gaa* double-knockout livers show reduced glycogen content as compared to *Gaa* (single-) knockout mice [33]. These mouse models do not show hypoglycemia, likely because they exhibit intact hepatic G6PC activity and functional glycogen degradation through PYGL. Taken together, we hypothesize that a reduction of STBD1-and GAA-mediated glycogen degradation in lysosomes may have contributed to (exacerbated) glycogen accumulation in S4048-infused mice treated with shSCR and shChREBP. Reduced lysosomal glycogen breakdown may furthermore have limited G6PC-independent glucose production [23], thereby contributing to lower blood glucose levels in response to S4048. Upon combined S4048/shChREBP treatment, G6PC-independent glucose production by the liver is likely even more strongly suppressed by concomitant reduction in STBD1/GAA and induction of GCK, resulting in enhanced cytosolic phosphorylation of free glucose produced through lysosomal glycogen breakdown [23]. As such, the further reduction in blood glucose levels observed in hepatic ChREBP-attenuated mice treated with S4048 may represent a trade-off for ChREBP-mediated homeostasis of intrahepatic phosphate esters and glycogen.

Intrahepatic G6P has previously been shown to suppress *Gck* mRNA levels, while hepatic ChREBP transcriptionally regulates the inhibitory GCK-binding protein GCKR [10,13,38,39]. Consistent with Arden et al. [10], we found that *Gck* mRNA levels were reduced by S4048 in shSCR-treated mice. Interestingly, we also observed that mRNA levels of *Gck*, rather than *Gckr* [4,40], were affected by shChREBP treatment. In line with these data, hepatic ChREBP overexpression reduces hepatic *Gck* mRNA levels (gse61576) [35], while ChREBP knockout mice show increased *Gck* mRNA levels [11]. Previous work showed that MLX, the binding partner of ChREBP, but not ChREBP itself, mediates glucose-mediated *Gck* repression in hepatocytes and mouse liver [10,13,41]. Besides increased *Gck* mRNA levels, shChREBP-treated mice showed elevated GCK protein levels, as well as increased cytosolic GCK/GCKR protein ratios. Fructose 1-phosphate (F1P), a substrate of Aldolase B (ALDOB), is known to enhance cytosolic glucokinase translocation and, consequently, activity by inhibiting the interaction between GCK and GCKR [42]. Given that *Aldob* is a well-established ChREBP target gene [13,38], it is conceivable that reduced ALDOB expression in shChREBP-treated mice (Supplementary Figure 3) increased F1P levels and a consequent increase in cytosolic GCK/GCKR levels and GCK flux. The metabolomics method used does not allow to specifically quantify F1P levels, but rather provides total hexose phosphate signals which likely include glucose 1-phosphate and fructose 6-phosphate (F6P) as well. Importantly, although higher F1P levels may have contributed to increased hexose phosphate levels in shChREBP-treated mice, F6P is known to exert an opposite effect on the GCK-GCKR interaction and GCK activity as compared to F1P [42,43]. Altogether, elucidation of the molecular mechanisms underlying *Gck* induction and increased cytosolic GCK/GCKR levels in response to hepatic ChREBP knockdown warrant further investigation. Finally, increased GCK levels and -flux in shChREBP-treated mice may have contributed to the further reduction in blood glucose levels and more excessive glycogen storage in shChREBP-treated mice infused with S4048.

Several study limitations warrant discussion. First, as steady-state isotope enrichment was achieved after 240 min, metabolic fluxes were calculated during the final 2 h of the infusion experiment. These fluxes do not necessarily reflect initial adaptations in hepatic glucose and glycogen metabolism that occur in response to S4048 infusion, and the role of ChREBP herein. We therefore cannot exclude that initial flux adaptations which may have contributed to the observed changes in metabolite levels are being left unnoticed because at the time isotopic steady state enrichments were reached, new steady states in fluxes had been reached. Second, as the applied method relies on a flux balance model, i.e., the sum of calculated fluxes toward G6P (glucokinase, glycogen phosphorylase, gluconeogenesis) equals the sum of fluxes away from G6P (glucose-6-phosphatase and glycogen synthase), only fluxes included in the model could be quantified. Importantly, glycolysis, a third flux away from G6P, is not considered in the model, due to the impossibility of time-resolved repetitive sampling of glycolytic intermediates *in vivo*. It is therefore conceivable that, under consistent G6P levels, the estimated fluxes toward G6P are in reality higher, and GCK flux, for instance, was underestimated. Notably, m+3/m+6 ratios in blood- and UDP-glucose, a measure for infused glucose that was first used in glycolysis and subsequently redirected towards (UDP)-glucose, were increased upon shChREBP treatment. This indicates an increased contribution of glycolytic intermediates for glucose and glycogen production in response to shChREBP. These findings are in line with ChREBP being a key regulator of downstream glycolysis via transcriptional control of pyruvate kinase. Moreover, m+3/m+6 ratios in blood and UDP-glucose were reduced upon S4048 infusion, indicating the lower contribution of glycolytic intermediates for glucose and glycogen production, consistent with an increased glycolytic flux in acute hepatic GSD Ib [44]. Regarding the flux balance model assuming stable G6P levels: as hepatic G6P accumulated during the course of S4048 infusion, the current model may have underestimated the fluxes toward G6P and/or overestimated the fluxes away from G6P. Third, the flux balance model does not account for G6PC-independent glucose production via AGL- and/or GAA-mediated glycogenolysis. With the applied model, these contributions are accounted for as PYGL and G6Pase fluxes. In addition, as a result of enhanced GCK flux upon shChREBP treatment, a larger fraction of such intrahepatic free glucose produced through AGL and/or GAA may be directly phosphorylated to G6P as compared to shSCR conditions. Such potential excess G6P synthesis in shChREBP-treated mice is assigned to the PYGL flux. In order to precisely assess the contribution of AGL- and/or GAA-mediated alternative glycogen breakdown under these conditions, complementary studies in *e.g., Agl*/*Gaa* double-knockout mice should be performed. Fourth, in this study outcomes on gene, protein and metabolite levels obtained from one animal cohort were related to flux data obtained upon isotope infusion in a second animal cohort. Although we aimed to administer tracer amounts of the labeled metabolites, it should be noted that blood glucose concentrations were higher in the isotope-infused versus non-isotope cohort. This increase may be explained by gluconeogenic precursors infused during the isotope infusion experiment increasing hepatic glucose production and glycemia. Fifth, the statistical power was likely compromised due to lack of urine samples from a few mice, especially shChREBP-treated mice infused with S4048. This limited sample size may, for example, explain that only a tendency towards a statistically significant increase in GCK flux was observed in shChREBP- *vs.* shSCR-treated S4048-infused mice.

In conclusion, this study extends the current perspective on the role of ChREBP in regulating hepatic glucose and glycogen metabolism. Interestingly, our work shows increased cytosolic GCK levels and enhanced GCK flux upon moderate hepatic ChREBP knockdown, while GCKR levels remain unaltered. Furthermore, ChREBP knockdown also enhances glycogen cycling in acute hepatic GSD Ib, despite a reduction in GYS2 and PYGL levels. Secondly, the findings indicate a dominant effect of metabolite and/or hormone-dependent regulation of glycogen synthesis and -breakdown fluxes over the regulation by enzyme levels under these conditions. Finally, our results suggest that ChREBP controls STBD1 levels and GAA enzyme activity, two key mediators of lysosomal glycogen breakdown, which requires further investigation. Integrating our results, we propose that excess hepatic G6P accumulated in response to glycolysis inhibition in ChREBP-normalized GSD Ib mice flows over to liver glycogen via glycogen synthase. The concomitant reduction of lysosomal glycogen breakdown in ChREBP-normalized GSD Ib mice further increases hepatic glycogen content, while reducing alpha glucosidase-dependent glucose production. The latter results in more pronounced hypoglycemia in ChREBP-normalized hepatic GSD Ib mice, which in turn increases the glucagon/insulin ratio, that subsequently enhances the flux through hepatic glycogen phosphorylase. Overall, our study confirms the importance of ChREBP in balancing metabolic fluxes responsible for controlling hepatic glycogen content and blood glucose levels in metabolically compromised states [1].

## Declaration of competing interest

The authors declare that they have no known competing financial interests or personal relationships that could have appeared to influence the work reported in this paper.

## Data Availability

Data will be made available on request.

## References

[1] Agius L., Chachra S.S., Ford B.E. (2020). The protective role of the carbohydrate response element binding protein in the liver: the metabolite perspective. Front Endocrinol (Lausanne).

[2] Dentin R. (2012). Glucose 6-phosphate, rather than xylulose 5-phosphate, is required for the activation of ChREBP in response to glucose in the liver. J Hepatol.

[3] Kabashima T., Kawaguchi T., Wadzinski B.E., Uyeda K. (2003). Xylulose 5-phosphate mediates glucose-induced lipogenesis by xylulose 5-phosphate-activated protein phosphatase in rat liver. Proc Natl Acad Sci USA.

[4] Arden C., Tudhope J., Susan, Petrie L., John Al-Oanzi H., Ziad (2012). Fructose 2,6-bisphosphate is essential for glucose-regulated gene transcription of glucose-6-phosphatase and other ChREBP target genes in hepatocytes. Biochem J.

[5] Grefhorst A., Schreurs M., Oosterveer H., Maaike, Cortés A., Victor (2010). Carbohydrate-response-element-binding protein (ChREBP) and not the liver X receptor α (LXRα) mediates elevated hepatic lipogenic gene expression in a mouse model of glycogen storage disease type 1. Biochem J.

[6] Dentin R., Benhamed F., Hainault I., Fauveau Vr, Foufelle F., Dyck J.R.B. (2006). Liver-specific inhibition of ChREBP improves hepatic steatosis and insulin resistance in ob/ob mice. Diabetes.

[7] Eissing L., Scherer T., Tödter K., Knippschild U., Greve J.W., Buurman W.A. (2013). De novo lipogenesis in human fat and liver is linked to ChREBP-β and metabolic health. Nat Commun.

[8] Kursawe R., Caprio S., Giannini C., Narayan D., Lin A., D'Adamo E. (2013). Decreased transcription of ChREBP-α/β isoforms in abdominal subcutaneous adipose tissue of obese adolescents with prediabetes or early type 2 diabetes: associations with insulin resistance and hyperglycemia. Diabetes.

[9] Lei Y., Hoogerland J.A., Bloks V.W., Bos T., Bleeker A., Wolters H. (2020). Hepatic carbohydrate response element binding protein activation limits nonalcoholic fatty liver disease development in a mouse model for glycogen storage disease type 1a. Hepatology.

[10] Arden C., Petrie J.L., Tudhope S.J., Al-Oanzi Z., Claydon A.J., Beynon R.J. (2011). Elevated glucose represses liver glucokinase and induces its regulatory protein to safeguard hepatic phosphate homeostasis. Diabetes.

[11] Iizuka K., Bruick R.K., Liang G., Horton J.D., Uyeda K. (2004). Deficiency of carbohydrate response element-binding protein (ChREBP) reduces lipogenesis as well as glycolysis. Proc Natl Acad Sci USA.

[12] Shi J.-H., Lu J.-Y., Chen H.-Y., Wei C.-C., Xu X., Li H. (2020). Liver ChREBP protects against fructose-induced glycogenic hepatotoxicity by regulating L-type pyruvate kinase. Diabetes.

[13] Poungvarin N. (2015). Genome-wide analysis of ChREBP binding sites on male mouse liver and white adipose chromatin. Endocrinology.

[14] Sherigar J.M., Castro J.D., Yin Y.M., Guss D., Mohanty S.R. (2018). Glycogenic hepatopathy: a narrative review. World J Hepatol.

[15] Liu Q., Li J., Zhang W., Xiao C., Zhang S., Nian C. (2021). Glycogen accumulation and phase separation drives liver tumor initiation. Cell.

[16] Dong X., Wang F., Liu C., Ling J., Jia X., Shen F. (2021). Single-cell analysis reveals the intra-tumor heterogeneity and identifies MLXIPL as a biomarker in the cellular trajectory of hepatocellular carcinoma. Cell Death Discovery.

[17] Wang H., Dolezal J.M., Kulkarni S., Lu J., Mandel J., Jackson L.E. (2018). Myc and ChREBP transcription factors cooperatively regulate normal and neoplastic hepatocyte proliferation in mice. J Biol Chem.

[18] Teryukova N.P., Malkova V.V., Sakhenberg E.I., Ivanov V.A., Bezborodkina N.N., Snopov S.A. (2018). On reprogramming of tumor cells metabolism: detection of glycogen in the cell lines of hepatocellular origin with various degrees of dedifferentiation. Cytotechnology.

[19] Ribback S., Che L., Pilo M.G., Cigliano A., Latte G., Pes G.M. (2018). Oncogene-dependent addiction to carbohydrate-responsive element binding protein in hepatocellular carcinoma. Cell Cycle.

[20] Wang H., Lu J., Alencastro F., Roberts A., Fiedor J., Carroll P. (2022). Coordinated cross-talk between the myc and mlx networks in liver regeneration and neoplasia. Cell Mol Gastroenterol Hepatol.

[21] Rutten M.G.S., Derks T.G.J., Huijkman N.C.A., Bos T., Kloosterhuis N.J., Kolk K.C.W.A. (2021). Modeling phenotypic heterogeneity of glycogen storage disease type 1a liver disease in mice by somatic CRISPR/CRISPR-associated protein 9–mediated gene editing. Hepatology.

[22] Lei Y., Hu Q., Gu J. (2020). Expressions of carbohydrate response element binding protein and glucose transporters in liver cancer and clinical significance. Pathol Oncol Res.

[23] Hijmans B.S., Boss A., Van Dijk T.H., Soty M., Wolters H., Mutel E. (2017). Hepatocytes contribute to residual glucose production in a mouse model for glycogen storage disease type Ia. Hepatology.

[24] Oosterveer M.H., Schoonjans K. (2014). Hepatic glucose sensing and integrative pathways in the liver. Cell Mol Life Sci.

[25] Rajas F., Gautier-Stein A., Mithieux G. (2019). Glucose-6 phosphate, a central hub for liver carbohydrate metabolism. Metabolites.

[26] Van Dijk T.H., Van Der Sluijs F.H., Wiegman C.H., Baller J.F.W., Gustafson L.A., Burger H.-J. (2001). Acute inhibition of hepatic glucose-6-phosphatase does not affect gluconeogenesis but directs gluconeogenic flux toward glycogen in fasted rats. J Biol Chem.

[27] Bandsma R.H.J., Van Dijk T.H., Harmsel A.T., Kok T., Reijngoud D.-J., Staels B. (2004). Hepatic de Novo synthesis of glucose 6-phosphate is not affected in peroxisome proliferator-activated receptor α-deficient mice but is preferentially directed toward hepatic glycogen stores after a short term fast. J Biol Chem.

[28] Lee W.-N.P., Byerley L.O., Bergner E.A., Edmond J. (1991). Mass isotopomer analysis: theoretical and practical considerations. Biol Mass Spectrom.

[29] Bergmeyer H.U., Gawehn K. (1978).

[30] Schomakers B.V., Hermans J., Jaspers Y.R.J., Salomons G., Vaz F.M., van Weeghel M. (2022). Polar metabolomics in human muscle biopsies using a liquid-liquid extraction and full-scan LC-MS. STAR Protoc.

[31] Okumiya T., Keulemans J.L., Kroos M.A., Van der Beek N.M., Boer M.A., Takeuchi H. (2006). A new diagnostic assay for glycogen storage disease type II in mixed leukocytes. Mol Genet Metabol.

[32] Oosterveer M.H., Grefhorst A., Van Dijk T.H., Havinga R., Staels B., Kuipers F. (2009). Fenofibrate simultaneously induces hepatic fatty acid oxidation, synthesis, and elongation in mice. J Biol Chem.

[33] Sun T., Yi H., Yang C., Kishnani P.S., Sun B. (2016). Starch binding domain-containing protein 1 plays a dominant role in glycogen transport to lysosomes in liver. J Biol Chem.

[34] Bouskila M., Hunter R.W., Ibrahim A.F.M., Delattre L., Peggie M., Van Diepen J.A. (2010). Allosteric regulation of glycogen synthase controls glycogen synthesis in muscle. Cell Metabol.

[35] Benhamed F., Denechaud P.-D., Lemoine M., Robichon C., Moldes M., Bertrand-Michel J. (2012). The lipogenic transcription factor ChREBP dissociates hepatic steatosis from insulin resistance in mice and humans. J Clin Invest.

[36] Metzendorf C., Wineberger K., Rausch J., Cigliano A., Peters K., Sun B. (2020). Transcriptomic and proteomic analysis of clear cell foci (CCF) in the human non-cirrhotic liver identifies several differentially expressed genes and proteins with functions in cancer cell biology and glycogen metabolism. Molecules.

[37] Kyriakoudi S., Theodoulou A., Potamiti L., Schumacher F., Zachariou M., Papacharalambous R. (2022). Stbd1-deficient mice display insulin resistance associated with enhanced hepatic ER-mitochondria contact. Biochimie.

[38] Ma L., Robinson L.N., Towle H.C. (2006). ChREBP•Mlx is the principal mediator of glucose-induced gene expression in the liver. J Biol Chem.

[39] Towle H.C., Kaytor E.N., Shih H.M. (1997). Regulation of the expression of lipogenic enzyme genes by carbohydrate. Annu Rev Nutr.

[40] Kim M.-S., Krawczyk S.A., Doridot L., Fowler A.J., Wang J.X., Trauger S.A. (2016). ChREBP regulates fructose-induced glucose production independently of insulin signaling. J Clin Invest.

[41] Yu A., Yu P., Zhu Y., Zhu R., Sun R., Ye D. (2023 Oct). Glucose-induced and ChREBP: MLX-mediated lipogenic program promotes hepatocellular carcinoma development. Oncogene.

[42] Davies D.R., Detheux M., Schaftingen E. (1990). Fructose 1-phosphate and the regulation of glucokinase activity in isolated hepatocytes. Eur J Biochem.

[43] Veiga-Da-Cunha M., Van Schaftingen E. (2002). Identification of fructose 6-phosphate- and fructose 1-Phosphate-binding residues in the regulatory protein of glucokinase. J Biol Chem.

[44] Bandsma R.H.J., Wiegman C.H., Herling A.W., Burger H.-J., ter Harmsel A., Meijer A.J. (2001). Acute inhibition of glucose-6-phosphate translocator activity leads to increased de novo lipogenesis and development of hepatic steatosis without affecting VLDL production in rats. Diabetes.

