# Supplementary material for: Hepatic ChREBP orchestrates intrahepatic carbohydrate metabolism to limit hepatic glucose 6-phosphate and glycogen accumulation in a mouse model for acute Glycogen Storage Disease type Ib
Source: Mol Metab. 2023 Nov 22;79:101838. doi: 10.1016/j.molmet.2023.101838 (PMC10716006; doi:10.1016/j.molmet.2023.101838)
Supplement: Multimedia component 1 [file mmc1.docx]

**Supplementary figure 1**


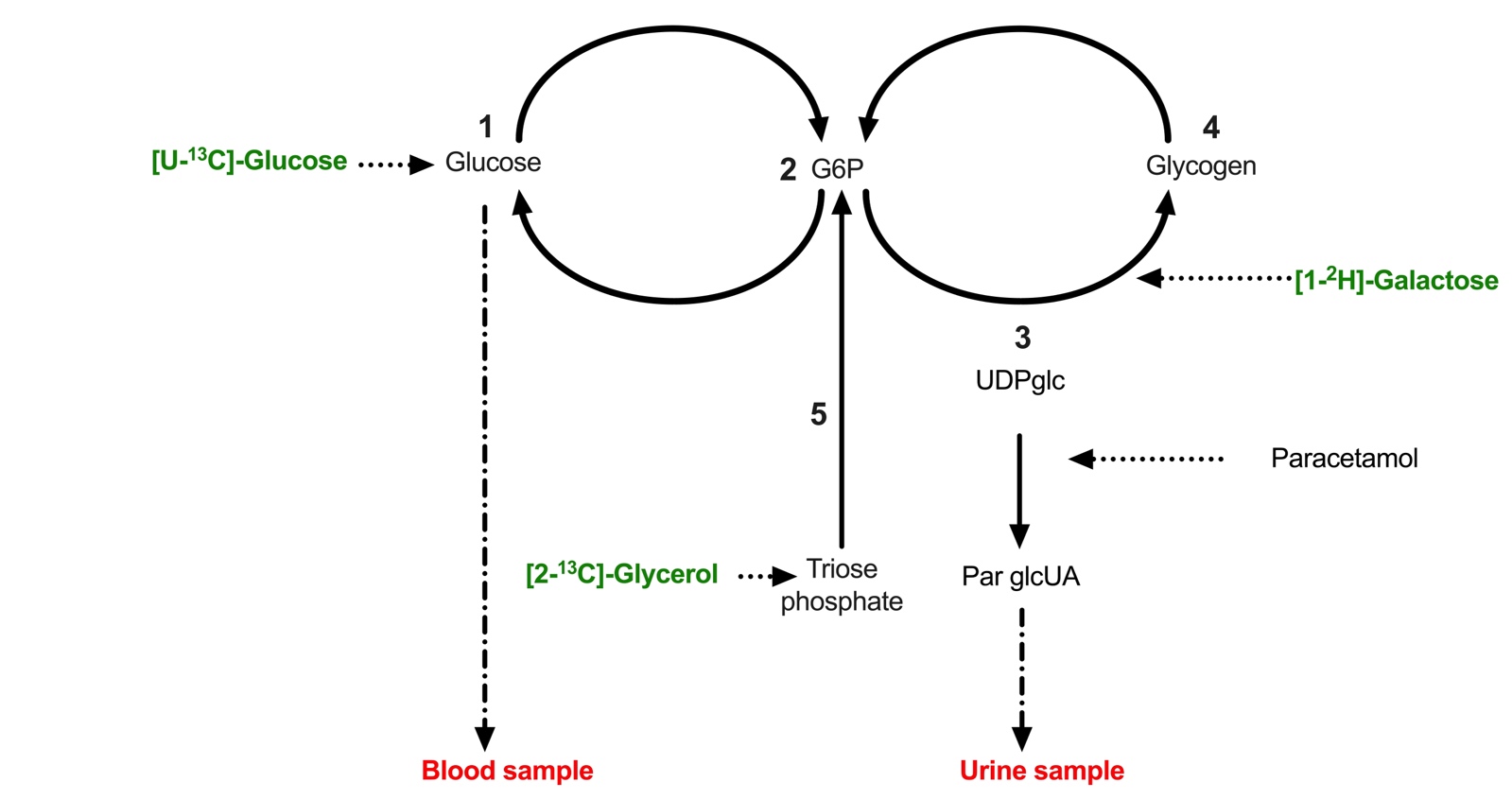


Schematic representation of stable isotope infusion (green font), and sample collection (red font).

G6P, glucose 6-phosphate; UDPglc, UDP glucose; Par glcUA, Paracetamol glucuronic acid. Numbers 1-5 indicate the metabolite pools used for flux parameter calculations outlined in the table below.

| Equations used for calculation of parameters and fluxes presented in Figure 2 and Table 3. | |
| --- | --- |
| **Primary isotope parameters** | |
| d(glc)  Isotope dilution | $\frac{\left( m_{\mathrm{glc}}^{+6} \right)}{\left( m_{glc,inf}^{+6} \right)}$ |
| d(UDPglc)  Isotope dilution | $\frac{(m_{\mathrm{UDPglc}}^{+1} )}{{(m}_{UDPglc,inf}^{+1})}$ |
| c(glc)  Isotope exchange; fractional contribution of blood glucose in UDP-glucose | $\frac{\left( m_{\mathrm{UDPglc}}^{+6} \right)}{\left( m_{\mathrm{glc}}^{+6} \right)}$ |
| c(UDPglc)  Isotope exchange; fractional contribution of UDP-glucose in blood glucose | $\frac{\left( m_{\mathrm{glc}}^{+1} \right)}{\left( m_{\mathrm{UDPglc}}^{+1} \right)}$ |

| Total glucose turnover rate  Rd(glc) | $\frac{\left( m_{glc,inf}^{+6} \right)}{\left( m_{\mathrm{glc}}^{+6} \right)}I_{glc}$ |
| --- | --- |
| Endogenous glucose production rate Ra(glc) | Rd(glc) - $I_{\mathrm{glc}}$ |
| Metabolic glucose clearance rate | $\frac{Rd(glc)}{\left( blood glucose level \right)}$ |
| UDP glucose turnover rate Rd(UDPglc) | $\frac{\left( m_{UDPglc,inf}^{+1} \right)}{\left( m_{\mathrm{UDPglc}}^{+1} \right)} I_{\mathrm{gal}}$ |
| Rate of appearance of UDPglc Ra(UDPglc) | Rd(UDPglc) – $I_{\mathrm{gal}}$ |
| **Flux parameters** | |
| Glucose cycling (Glucose-G6P recycling)  r(1,1) | $\frac{c(glc)}{\left( 1- c(glc) \right)}\mathrm{Ra}\left( \mathrm{glc} \right)$ |
| Total endogenous glucose production rate Ra_total_(glc) | Ra(glc) + r(1,1) |
| Flux from blood glucose to UDPglc  r(3,1) | c(glc) * Rd(UDPglc) |
| Glucokinase flux* | r(3,1) + r(1,1) |
| Fractional contribution of gluconeogenesis (F16P) in blood glucose; c(1,5) | $\frac{m_{\mathrm{glc}}^{+2}}{m_{F16P}^{+2}}$ |
| Flux from newly synthesized F16P to blood glucose, r(1,5) | c(1,5) * Rd(glc) |
| Fractional contribution of gluconeogenesis (F16P) in UDPglc, c(3,5) | $\frac{m{+2\ldots\ldots\ldots.. \atop\mathrm{UDPglc}}}{m{+2 \atop F16P}\ldots..}$ |
| r(1,5)_indirect_ (route from F16P via UDPglc to blood glucose) | c(3,5) * r(1,3) |
| r(1,5)_direct_ | r(1,5) – r(1,5)_indirect_ |
| Flux from glycogen to blood glucose  r(1,4) | Ra(glc) – r(1,5)_direct_ |
| Glucose 6-phosphatase flux | r(1,5)_direct_ + r(1,4) + r(1,1) |
| Glycogen synthase flux | Ratotal(UDPglc) + I_gal_ |
| UDPglc recycling rate, r(3,3) | $\frac{c(UDPglc)}{\left( 1- c(UDPglc) \right)} *Ra\left( \mathrm{UDPglc} \right)$ |
| Flux of F16P to UDPglc, r(3,5) | c(3,5) * Rd(UDPglc) |
| r(3,5)_indirect_ (route from F16P via blood glucose to UDPglc) | c(1,5) * r(3,1) |
| r(3,5)_direct_ | r(3,5) - r(3,5)_indirect_ |
| Flux from glycogen to UDPglc  r(3,4) | Rd(UDPglc) - r(3,5)_direct_ - r(3,1) |
| Glycogen phosphorylase flux | r(1,4)+ r(3,4) + r(3,3) |
| Gluconeogenesis flux | r(1,5)_direct_ + r(3,5)_direct_ |

** The glucokinase flux represents the sum of fluxes through all hexokinase isoenzymes*

BG, Blood Glucose; Ra (glc), Rate of appearance of labelled glucose; Ra (UDPglc), Rate of appearance of labelled UDP-glucose; UDPglc, UDP-glucose.

$m_{\mathrm{glc}}^{+x}$ : measured isotopologue enrichment of x-labelled glucose in blood glucose

$m_{\mathrm{UDPglc}}^{+x}$ : measured isotopologue enrichment of x-labelled glucose as measured in the glucose part of UDPglc (urine)

$m_{glc,inf}^{+x}$ : measured isotopologue enrichment of x-labelled glucose in infusate

$m_{UDPglc,inf}^{+x}$ : measured isotopologue enrichment of x-labelled glucose as measured in the glucose part of UDPglc in infusate

$I_{\mathrm{glc}}$: glucose infusion rate (in μmol.kg^-1^.min^-1^)

$I_{\mathrm{gal}}$: galactose infusion rate (in μmol.kg^-1^.min^-1^)

$m_{F16P}^{+2}$ : theoretical isotopologue enrichment of newly synthesized doubly-labelled F16P as calculated by MIDA

Rate of disposal (Rd) = Rate of appearance (Ra) + Rate of infusion (I)

At a steady state Rd = Ra

Fractional contribution of the tracer, C = (I/Rd) = I/(Ra + I)
Numbers 1-5 indicate the metabolite pools used for flux parameter calculations presented in schematic respresentation.

These equations are derived from:

Van Dijk TH, Van Der Sluijs FH, Wiegman CH, Baller JFW, Gustafson LA, Burger H-J, et al.

Acute Inhibition of Hepatic Glucose-6-phosphatase Does Not Affect Gluconeogenesis but Directs Gluconeogenic Flux toward Glycogen in Fasted Rats.

Journal of Biological Chemistry. 2001;276(28):25727-35.


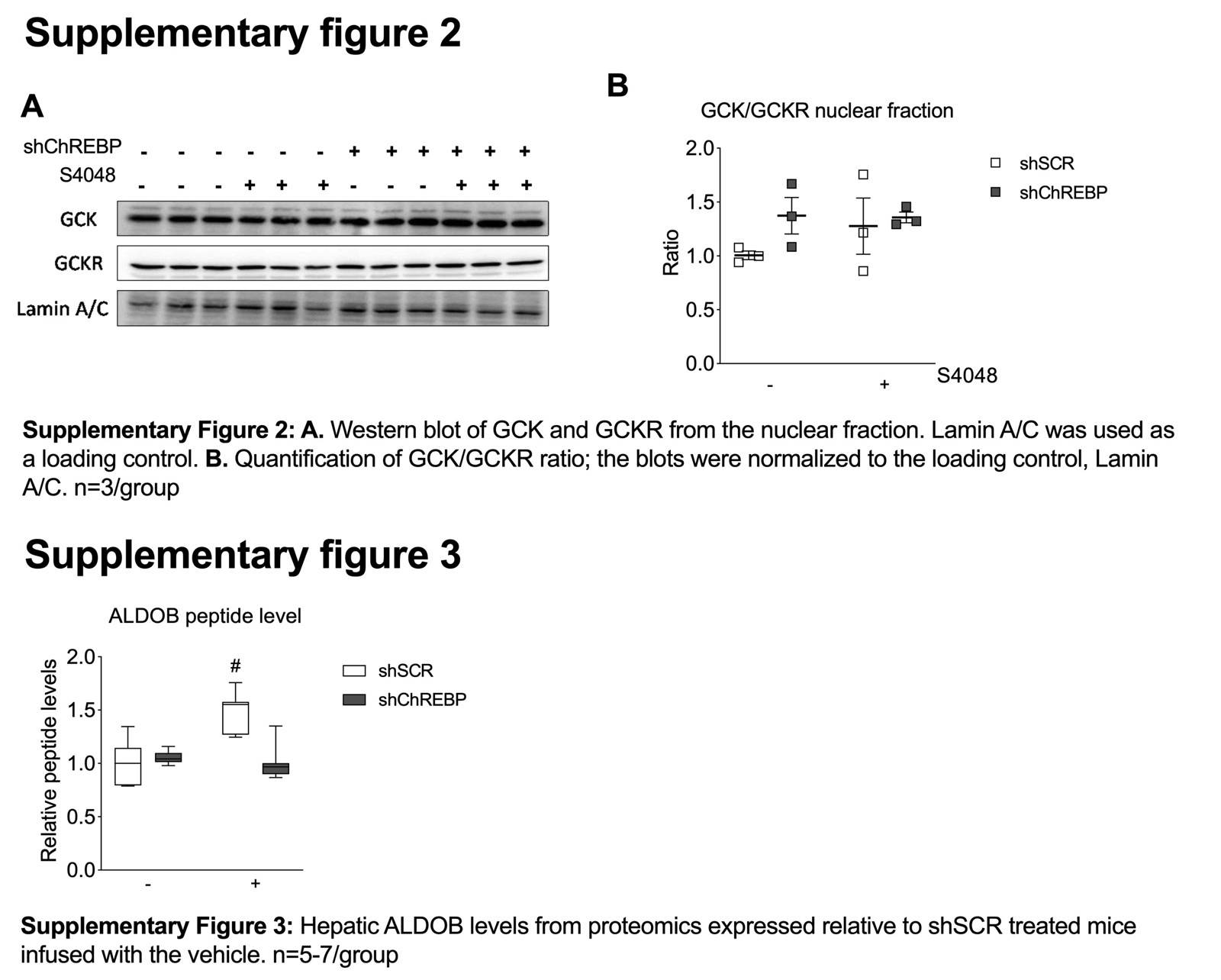


**Supplementary Tables**

Table S1: Primer/probe sequences used for qPCR analysis

| *Agl* | 5’CTATCCCGCTCGGGTAACT 3’  5’AGAGTTCGAATCCTCCAGAAGCCA 3’ |
| --- | --- |
| *Beta-Actin* | 5’CACTGTCGAGTCGCGTCC 3’  5’TCATCCATGGCGAAACTGGTG 3’ |
| *Chrebp-α* | 5’CGACACTCACCCACCTCTTC 3’  5’TTGTTCAGCCGGATCTTGTC 3’  CCTGGCTTACAGTGGCAAGCTGGTCTC (probe) |
| *Chrebp-β* | 5’TCTGCAGATCGCGTGGAG 3’  5’CTTGTCCCGGCATAGCAAC 3’  CTCAGTGGCAAGCTGGTCTCTCCCA (probe) |
| *G6pc* | 5’TTCCCTGTCACCTGTGAGACC 3’  5’ACATAGTATACACCTGCTGCGCC 3’  CAGGAAGTCCCTCTGGCCATGCC (probe) |
| *Gaa* | 5’CTTCAAGATCAAAGATCCTGCTAGTAAG 3’  5’TGAGAATTCCACGCTGTAAAGTG 3’ |
| *Gck* | 5’GCGGAGATGCTCTTTGACTAC 3’  5’TGTCTATGTCTTCGTGCCTTAC 3’ |
| *Gckr* | 5’CAACTCCAAGCTCTTCTGGAG 3’  5’CCTAACAACCTCACAGACTGAAG 3’ |
| *Gys2* | 5’GCTCTCCAGACGATTCTTGCA 3’  5’GTGCGGTTCCTCTGAATGATC 3’ |
| *Pklr* | 5’CGTTTGTGCCACACAGATGCT 3’  5’CATTGGCCACATCGCTTGTCT 3’ |
| *Pygl* | 5’GAAGGAGGCAAACGGATCAAC 3’  5’TCACGATGTCCGAGTGGATCT 3’ |
| *Slc37a4* | 5’GAGGCCTTGTAGGAAGCATTG 3’  5’CCATCCCAGCCATCATGAGTA 3’  CTCTGTATGGGAACCCTCGCCACG (probe) |
| *Stbd1* | 5’TCTCCAAAGCAGAGCATCTTCG 3’  5’ACCCAGTCTGCTCCAACATT 3’ |

Table S2: Antibodies used for immunoblot analysis

| Antibody | Dilution | Source | Reference |
| --- | --- | --- | --- |
| GCK | 1:1000 | Rabbit | Sc-17819 |
| GCKR | 1:1000 | Rabbit | Sc-74544 |
| HSP90 | 1:1000 | Rabbit | CST #4874 |
| Lamin A/C | 1:1000 | Rabbit | CST #4777 |
| Anti-rabbit HRP-conjugated (secondary) | 1:2000 | Goat | Biorad #1706515 |

Table S3: Peptide sequences of the labeled standards used for proteomics analysis

| Protein | Target peptide |
| --- | --- |
| AGL | VSLDPHAQVAVGILR |
| ALDOB | ALQASALAAWGGK |
| G6PC | GLGVDLLWTLEK |
| GAA | WGYSSTAIVR |
| GCK | ITVGVDGSVYK |
| GYS2 | GADIFLESLSR |
| PKLR | STSIIATIGPASR |
| PYGL | TFAYTNHTVLPEALER |
| SLC37A4 | AGLSLYGNPR |
| STBD1 | DGLWSHSVFLPADTVVEWK |
